# Poor Access to Liver Transplantation and Survival of Children With Acute Liver Failure, Acute-on-chronic Liver Failure or Chronic Liver Disease

**DOI:** 10.1097/PG9.0000000000000318

**Published:** 2023-06-09

**Authors:** Emma Valeria Estrada-Arce, Renata Aguila-Cano, Juan Carlos Lona-Reyes, Laura Esther Flores-Fong, Elva Rivera-Chávez

**Affiliations:** From the * Pediatric Gastroenterology Service, Hospital Civil de Guadalajara “Dr. Juan I. Menchaca” Salvador Quevedo y Zubieta, Guadalajara, Jalisco, México; † Universidad de Guadalajara, University Center for Health Sciences; ‡ Pediatric Infectology Service, Hospital Civil de Guadalajara “Dr. Juan I. Menchaca”; § Universidad de Guadalajara, Tonalá University Center.

**Keywords:** end-stage liver disease, liver diseases, liver failure, acute

## Abstract

We describe the survival of children with acute liver failure (ALF), chronic liver disease (CLD), or acute-on-chronic liver failure (ACLF) with poor access to liver transplantation (LT). A retrospective cohort study of 42 patients <18 years of age was conducted in the Hospital Civil de Guadalajara “Dr. Juan I. Menchaca”. The median age was 76 months; 57.1% were female, 40.5% presented with ALF, 35.7% with CLD, and 23.8% with ACLF. Also, 38.1% (16/42) presented liver disease of unknown etiology. Death occurred in 45.2%; 14.3% were transferred to another hospital, and none received LT. Mortality in ALF, CLD, and ACLF was 76%, 0%, and 60%, respectively. In the survival analysis, within the first 20 months after diagnosis, the mortality rate was greater than 50% with ALF. The importance of having referral programs that perform liver transplantation is highlighted by the poor prognosis of the patients, despite conservative treatment.

**What Is Known**

- It is important to have healthcare systems with the capacity of providing liver transplantation for children diagnosed with acute liver failure, chronic or acute-on-chronic liver disease.

**What Is New**

- In a hospital located in western Mexico, survival in children with acute or acute-on-chronic liver failure was less than 25% during the first 2 years after diagnosis.

## INTRODUCTION

Acute Liver Failure (ALF) in children is a rapidly progressive condition with high morbidity and mortality (1). The most prevalent causes are infections, drug toxicity, or in association with immunological or metabolic diseases; however, a high percentage of cases have an undetermined etiology (2).

Chronic hepatitis is characterized by elevated transaminase levels for more than 3 months, and its main causes are infections, anatomical anomalies, autoimmune and metabolic diseases, and nonalcoholic steatohepatitis (3–5). An exception to this classification is infection by hepatitis B virus since it is classified as chronic when elevated transaminase levels and HBsAg persist for more than 6 months. In events where liver damage persists, the risk of developing liver fibrosis or cirrhosis is increased, with a higher occurrence of serious complications such as portal hypertension, ascites, bleeding from esophageal varices, hepatic encephalopathy, electrolyte imbalance, or hepatorenal syndrome (4,5).

No consistent definition of acute-on-chronic liver failure (ACLF) and the events that precipitate it are not clear. However, according to the Asian Pacific Association for the Study of the Liver, ACLF is an acute hepatic insult manifesting as jaundice (serum bilirubin ≥5 mg/dL) and coagulopathy (international normalized ratio [INR] ≥1.5 or prothrombin activity <40%) complicated within 4 weeks by clinical ascites and/or encephalopathy in a patient with previously diagnosed or undiagnosed chronic liver disease (CLD)/cirrhosis (6,7).

Although management consists of treating the specific etiology and its complications, in some cases the only intervention that significantly improves patient survival is liver transplantation (LT) (2). In developing countries, access to healthcare centers with the capacity of performing a liver transplant is limited. The objective of this study was to describe the survival of children with ALF, CLD, or ACLF in an environment where children have limited access to LT.

## METHODS

A retrospective cohort study was conducted at the Hospital Civil de Guadalajara “Dr. Juan I. Menchaca” (HCGJIM), in Guadalajara, Mexico. The institution is a tertiary referral center that includes the specialty of pediatric gastroenterology and an intensive care unit and is capable of providing dialysis. However, there is no hepatology service, and LT is not currently provided.

Charts of patients <18 years of age who were hospitalized and diagnosed with ALF, CLD, or ACLF between 2015 and 2020 in the pediatric gastroenterology service were reviewed. The diagnostic protocol in first-admission patients with ALF, CLD, or ACLF includes blood cytometry, liver enzyme and function tests, blood gas tests, serological tests for HIV, cytomegalovirus, Ebstein Barr virus, Herpes Simplex and hepatitis A, B, and C; serum levels of transferrin and ferritin, serum lipids, metabolic screen; hepatobiliary ultrasound; antinuclear, antismooth muscle, and liver and kidney microsomal type 1 antibodies (anti- LKM-1). Serum ammonia, ceruloplasmin, and urinary excretion of copper are conducted in outside laboratories. Liver biopsies are guided by ultrasound and are performed if the patient is hemodynamically stable. The sequence of studies is determined by the treating physician based on the main diagnostic consideration.

The patient’s general condition at the time of the study was recorded and included their survival rate, transfers to other hospitals, or occurrence of death. The age of the patient when ALF, CLD, or ACLF was diagnosed, the date of their last visit, demographic characteristics, clinical variables, nutritional status, complications, and biochemical parameters of their last visit were recorded.

## DEFINITIONS

*ALF*: biochemical evidence of acute liver damage and coagulopathy not corrected by vitamin K, with prothrombin time (PT) of 15–19.9 seconds or an INR of 1.5–1.9 if accompanied by encephalopathy. In events without encephalopathy and without known liver disease, the biochemical evidence of liver damage and the presence of PT >20 seconds or INR >2 were classified as ALF (8).

*CLD*: increased transaminase levels that persist ≥3 months or in the case of hepatitis B virus infection, persistence of HBsAg and elevated transaminase levels ≥6 months (9).

*ACLF*: acute hepatic insult manifesting as jaundice (serum bilirubin ≥5 mg/dL) and coagulopathy (INR ≥1.5 or prothrombin activity <40%) complicated within 4 weeks by clinical ascites and/or encephalopathy in a patient with previously diagnosed or undiagnosed CLD/cirrhosis (6,7,10).

*Cirrhosis*: hepatic fibrosis with distortion of the duct architecture and compression of the vasculature and biliary structures (5). The diagnosis was established by histology (Scheuer Scale, F4).

*Malnutrition*: in <2 years, weight-for-height <89%, and in ≥2 years body mass index below the 5th percentile.

## STATISTICAL ANALYSIS

Frequencies and percentages were estimated for qualitative variables and median with interquartile ranges (IQR) for quantitative variables. The chi-square test or Fisher’s exact test was used as hypothesis tests for proportions and the Mann–Whitney U or Kruskal–Wallis test for median comparisons.

Survival rate was estimated with 95% confidence intervals (95% CI) and Kaplan–Meier survival curves were performed with the log-rank test for the hypothesis contrast in the comparison of subgroups. A *P* value <0.05 was considered statistically significant.

We analyzed the discriminatory capacity of the Pediatric Chronic Liver Failure Sequential Organ Failure Assessment Score (pCLIF-SOFA) to predict death using the area under the curve (AUC) (11); an acceptable value of ≥0.70 was considered. IBM SPSS (Statistical Package for the Social Sciences) Statistics version 25 was used.

The pediatric end-stage liver disease/model for end-stage liver disease (PELD/MELD ) are prognostic scales useful for assessing liver function but do not assess other organs and systems, in this study, we analyzed the accuracy of the scores to predict mortality in patients with liver disease; PELD risk score was calculated for patients younger than 12 years old and MELD score for older than 12 years old. It used an online calculator from the Mayo Clinic (12,13) (https://www.mayoclinic.org/medical-professionals/transplant-medicine/calculators/meld-model/itt-20434705, https://www.mdcalc.com/calc/87/peld-score-pediatric-end-stage-liver-disease-younger-12).

The project was approved by the Ethics and Research Committee of the HCGJIM, with registration number 0494/21.

## RESULTS

Charts of 42 patients with a median age of 76 months (IQR, 147) were reviewed; 57.1% (24/42) were female. In total 40.5% (17/42) presented with ALF, 35.7% (15/42) with CLD, and 23.8% with ACLF (10/42). The etiology of liver disease was undetermined in 38.1% (16/42), the rest had anatomical anomalies (7/42), metabolic diseases (6/42), autoimmune hepatitis (6/42), infectious hepatitis (4/42), onco-hematological disease (2/42) and poisoning (1/42). At some point in the course of the disease, 88.1% presented with hepatomegaly (37/42), 45.2% with ascites (19/42), 42.9% with splenomegaly (18/42), 40.5% with encephalopathy (17/42), 26.2% with gastrointestinal bleeding (11/42), and 7.1% with cirrhosis (3/42).

The median values of serum laboratory tests at the last visit were: total bilirubin 8.8 mg/dl (IQR, 14.7), direct bilirubin 4.45 mg/dl (IQR, 9.1), alanine aminotransferase 100 U/L (IQR, 160.5), aspartate aminotransferase 105 U/L (IQR, 493.3), albumin 2.3 g/dl (IQR, 1.6), INR 1.63 (IQR, 0.94), creatinine 0.39 mg/dl (IQR, 4.8), sodium 135 mEq/L (IQR, 5.25), hemoglobin 9.8 g/dl (IQR, 3.3), platelets 95.8 thousand/ml (IQR, 128.6) and leukocytes 8.25 thousand/ml (IQR, 7.9). Table 1 shows the values depending on the syndrome in which each patient was classified.

**TABLE 1.** Comparison of clinical manifestations and laboratory results in children with acute liver failure, chronic liver disease and acute-on-chronic liver failure

		Acute hepatic failure n 17	Chronic liver disease n 15	Acute on chronic liver failure n 10	*P* *
Hepatomegaly	% (n)	82.4 (14)	100 (15)	80 (8)	0.2
Splenomegaly	% (n)	35.3 (6)	40 (6)	60 (6)	0.44
Ascites	% (n)	41.2 (7)	20 (3)	90 (9)	0.002
Encephalopathy	% (n)	70.6 (12)	0 (0)‡	50 (5)	<0,001
Cirrhosis	% (n)	0	6.7 (1)	20 (2)	0.15
Gastrointestinal bleeding	% (n)	23.5 (4)	20 (3)	40 (4)	0.51
Malnutrition	% (n)	47.1 (8)	13.3 (2)	50 (5)	0.26
Total bilirubin mg/dl	Median	11.6	0.76‡	13.1	0.001
Direct bilirubin mg/dl	Median	6.9	0.1‡	7.1	0.001
ALT U/L†	Median	99	48.0	109.7	0.27
AST U/L†	Median	107	60.0	203.8	0.09
Albumin gr/dl	Median	2.1	3.4‡	2.0	0.002
INR†	Median	1.9	1.1‡	2.02	<0.001
Creatinine mg/dl	Median	0.46	0.32	0.42	0.49
Sodium mEq/L	Median	136	136	133	0.09
Hemoglobin gr/dl	Median	9.0	11.9‡	9.3	0.003
Platelets miles/μl	Median	67	151	77.6	0.14
Leukocytes miles/μl	Median	10.8	5.3	6.5	0.26
Etiological diagnosis:		**n 17**	**n 15**	**n 10**	0.006
Anatomical etiology§	% (n/N)	5.9 (1/17)	13.3 (2/15)	40 (4/10)	
Metabolic diseases∥	% (n/N)	0	33.3 (5/15)	10 (1/10)	
Autoimmune hepatitis	% (n/N)	0	33.3 (5/15)	10 (1/10)	
Infectious hepatitis	% (n/N)	17.6 (3/17)	0	10 (1/10)	
Others^¶^	% (n/N)	11.8 (2/17)	6.7 (1/15)	0	
Cause not determined	% (n/N)	64.7 (11/17)	13.3 (2/15)	30 (3/10)	
PELD/MELD score	Median	20	0	28.4	<0.001
Deaths	% (n/N)	76.5 (13/17)	0	60 (6/10)	<0.001

Log Rank test *P* < 0.001.

INR, international normalized ratio; PELD/MELD score: pediatric end-stage liver disease/Model For end-stage liver disease.

*To contrast hypotheses in quantitative variables, the Kruskal-Wallis test was used and for qualitative variables chi-squared test.

†Alanine aminotransferase/aspartate aminotransferase.

‡Group in which difference was observed.

§Bile duct atresia (n 3), portal vein thrombosis (n. 3) Budd-Chiari syndrome (1).

∥Glycogenosis (n. 3), alpha 1 antitrypsin deficiency (n. 1), progressive familial intrahepatic cholestasis (n. 1), lysosomal acid lipase deficiency (n. 1).

^¶^Chemotherapy (n 1), hepatoblastoma (n 1), hemophagocytic syndrome (n. 1)

The median follow-up time for the patients was 2.9 months (IQR, 18.4). Death occurred in 45.2% (19/42, 95% CI, 30.7–60.4), 14.3% were transferred to another hospital unit (6/42) and the rest continued with their ambulatory follow-up. Mortality in patients with ALF, CLD, or ACLF was 76% (13/17), 0% (0/15), and 60% (6/10), respectively, and was significantly higher in patients with ALF and ACLF (*P* < 0.001).

**FIGURE 1.**
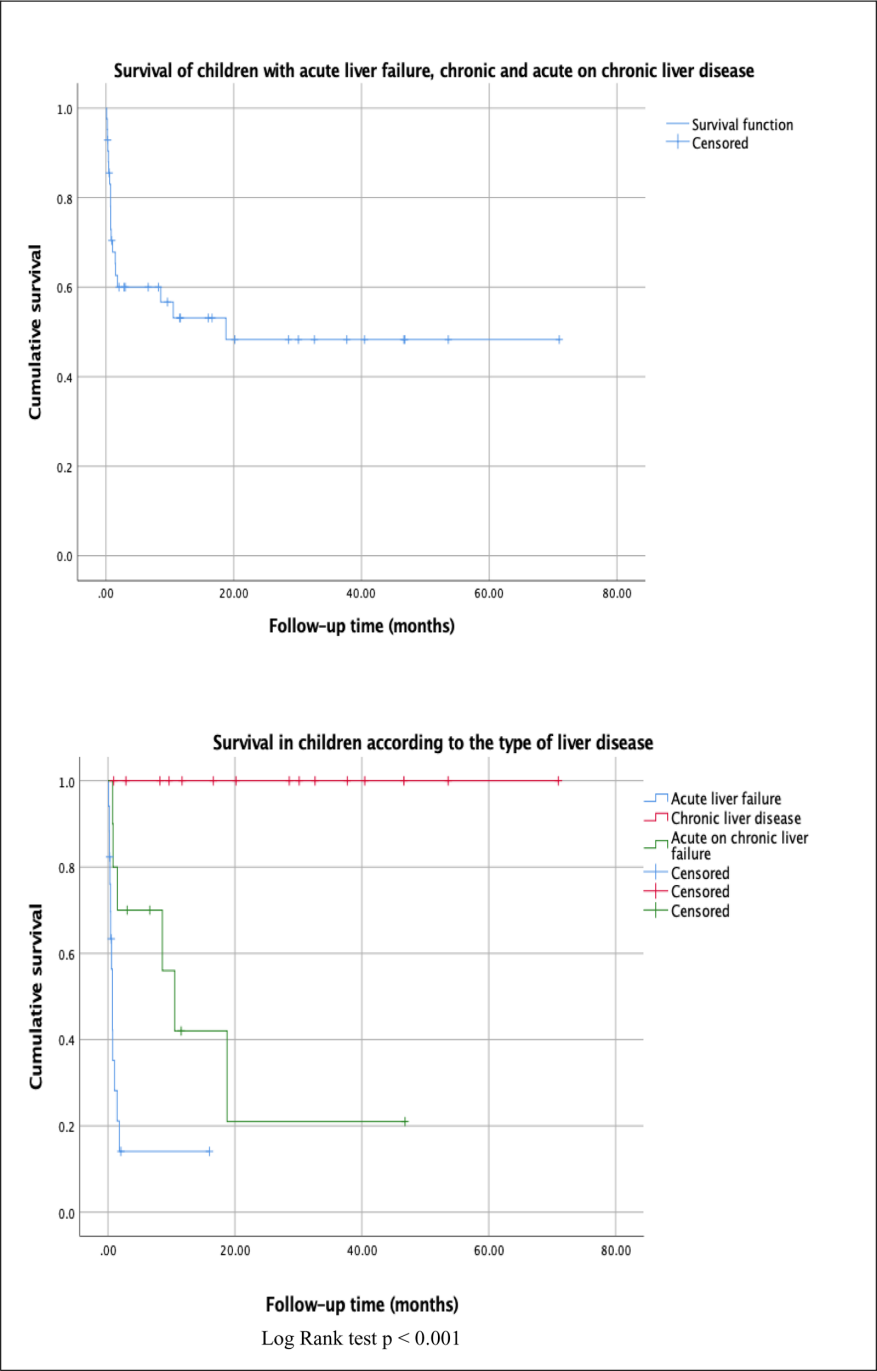
Survival of children with liver disease and according to the syndrome.

For the survival analysis, Kaplan–Meier curves were estimated for the total sample and as a function of the type of liver disease. We observed that in the first 20 months after the diagnosis of ALF, CLD, or ACLF a mortality rate greater than 50% was reached, being significantly higher in patients with ALF (Fig. 1). Six patients were referred to a hospital with a liver transplant unit, but none received LT, 1 continues under surveillance without achieving transplantation criteria, 2 died, and 3 were lost to follow-up.

We observed that the median in the PELD/MELD scores was 18 (IQR, 25.6) during their last assessment. The median of the scale for patients with ALF, CLD, or ACLF was 20, 0, and 28.45, respectively, showing a significant difference (*P* < 0.001). This instrument had an AUC of 0.84 (95% CI, 0.72–0.96) for the prediction of death, with a cutoff point ≥13, the scale showed sensitivity and specificity of 100% and 69.6%.

**FIGURE 2.**
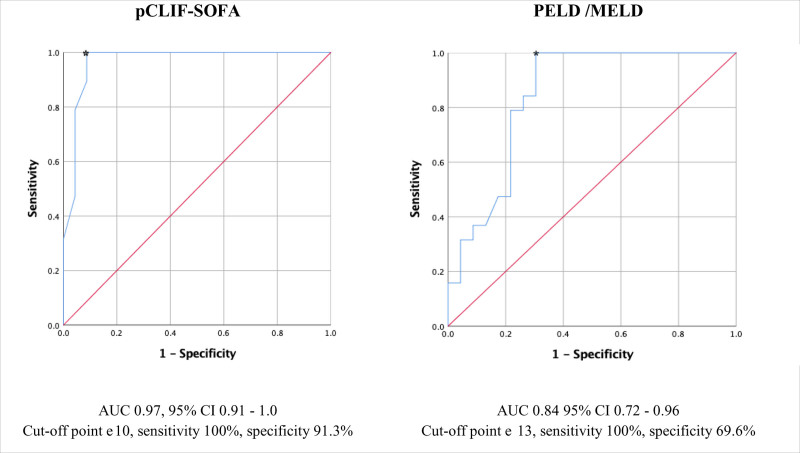
pCLIF-SOFA and PELD/MELD area under curve.

We also evaluated the pCLIF-SOFA, an instrument that has been validated in children that analyzes liver function in addition to respiratory, cardiovascular, renal, hematologic, and neurologic function. The maximum score is 24 and the higher the severity, the higher the score. When comparing the median scale scores between those who survived and those who died, we identified significant differences (4 vs. 13, *P* ≤ 0.001). The AUC was 0.97, 95% CI, 0.91–1.0. With a cutoff point of ≥10, the instrument presented sensitivity and specificity of 100% and 91.3%, while the negative and positive predictive values were 100% and 90.5% (Fig. 2).

## DISCUSSION

Our cohort revealed an overall mortality rate of 45.2% in children with ALF, CLD, or ACLF, however, it occurred more rapidly compared with other studies. One-year survival in children with ALF was less than 20%, a situation that highlights the importance of implementing healthcare centers with access to LT.

Bitar et al (14) identified the cause in 94% of children with early-onset and nontransplanted ALF, and found that the 1-year survival rate was 50%. Infants with galactosemia, tyrosinemia, and panhypopituitarism had the best prognosis. Different series have described survival with native liver of 24% to 60% (15). Our study found that children with ALF and ACLF presented with higher mortality. In these groups, an undetermined etiology was also more frequent, which suggests that not having an accurate diagnosis could influence the children’s prognosis. Other authors have reported that between 5% and 54% of children with ALF have an undetermined origin, while in chronic hepatobiliary disorders, the predominant causes are biliary atresia and storage diseases (4). In the HCGJIM’s patients, the majority of etiologies of ALF, CLD, or ACLF were undetermined (16/42), metabolic or autoimmune disease (12/42), and anatomical causes (7/42).

Lone et al (15) described that patients with unknown etiologies had worse outcomes. Alam et al (6) described poor survival at 28 days in children with ACLF with a mortality index of 25% and observed a worse prognosis in patients with unknown etiology.

We observed that ACLF had a 1-year survival rate of less than 25% and the clinical presentation of these patients included ascites (90%), hepatic encephalopathy (50%), and gastrointestinal bleeding (40%). In patients with CLD who are candidates for LT, it is important to refer the patient in a timely manner to care centers where this resource is available because the complications of the disease invariably progress to decompensation and death.

In this study, we observed that the pCLIF-SOFA scale showed adequate discriminatory capacity (AUC, 0.97). Similar to our results, Lal et al (16) described in children with ACLF that the SOFA scale showed adequate discrimination in the prediction of death (AUC, 0.95) and with a cutoff point of ≥6.5, they estimated sensitivity and specificity of 100% and 92%; with the same cutoff point, we observed sensitivity and specificity of 100% and 65.2%. On the other hand, Bolia et al (11) described for pCLIF-SOFA an AUC of 0.97 with a sensitivity and specificity of 94.9% and 91.5%, respectively. According to these results, we consider the pCLIF-SOFA scale to be a suitable instrument for predicting death in children with ALF, CLD, or ACLF.

In our study, the PELD/MELD prognostic scale was applied; the highest scores were recorded in children with ALF and ACLF. Jagadisan et al (17) evaluated the PELD scale for predicting death in children with ACLF and estimated an AUC of 0.91 and sensitivity and specificity of 100 and 83.3%, respectively.

In patients with timely and adequate treatment, the prognosis can improve significantly (18,19). Factors that negatively affect survival are the clinical condition of the patient, the severity of the condition, and access to specialized centers with the availability of living and/or cadaver donors for LT (20–22).
